# HSP27 and 70 expression in thymic epithelial tumors and benign thymic alterations: diagnostic, prognostic and physiologic implications

**DOI:** 10.1038/srep24267

**Published:** 2016-04-21

**Authors:** S. Janik, A. I. Schiefer, C. Bekos, P. Hacker, T. Haider, J. Moser, W. Klepetko, L. Müllauer, H. J. Ankersmit, B. Moser

**Affiliations:** 1 Department of Thoracic Surgery, Division of Surgery, Medical University Vienna, Austria; 2 Christian Doppler Laboratory for the Diagnosis and Regeneration of Cardiac and Thoracic Diseases, Medical University Vienna, Austria; 3 Clinical Institute of Pathology, Medical University Vienna, Austria; 4 University Clinic for Trauma Surgery, Medical University Vienna, Austria; 5 Departments of Dermatology and Venereology and Karl Landsteiner Institute of Dermatological Research, Karl Landsteiner University of Health Sciences, St. Pölten, Austria

## Abstract

Thymic Epithelial Tumors (TETs), the most common tumors in the anterior mediastinum in adults, show a unique association with autoimmune Myasthenia Gravis (MG) and represent a multidisciplinary diagnostic and therapeutic challenge. Neither risk factors nor established biomarkers for TETs exist. Predictive and diagnostic markers are urgently needed. Heat shock proteins (HSPs) are upregulated in several malignancies promoting tumor cell survival and metastases. We performed immunohistochemical staining of HSP27 and 70 in patients with TETs (n = 101) and patients with benign thymic alterations (n = 24). Further, serum HSP27 and 70 concentrations were determined in patients with TETs (n = 46), patients with benign thymic alterations (n = 33) and volunteers (n = 49) by using ELISA. HSPs were differentially expressed in histologic types and pathological tumor stages of TETs. Weak HSP tumor expression correlated with worse freedom from recurrence. Serum HSP concentrations were elevated in TETs and MG, correlated with clinical tumor stage and histologic subtype and decreased significantly after complete tumor resection. To conclude, we found HSP expression in the vast majority of TETs, in physiologic thymus and staining intensities in patients with TETs have been associated with prognosis. However, although interesting and promising the role of HSPs in TETs as diagnostic and prognostic or even therapeutic markers need to be further evaluated.

Thymic Epithelial Tumors (TETs) comprise thymomas, thymic carcinomas (TCs) and thymic neuroendocrine tumors (TNETs). Thymomas and TCs are the most common malignant neoplasms of the anterior mediastinum in adults and originate from thymic epithelial cells (TECs). TETs are rare malignancies with an incidence of 0.15 to 0.32 per 100.000 person-years^1^,^2^. Thymic malignancies are histologically classified according to the World Health Organization (WHO) classification, which distinguishes six main histopathologic types based on fundamental morphological differences: types A, AB, B1, B2, B3 thymomas and TCs^3^. TNETs are primary neuroendocrine tumors of the thymic gland that arise from thymic neuroendocrine cells and comprise less than 5% of TETs^4^,^5^. According to nomenclature of neuroendocrine tumors of the lung, TNETs are distinguished into well-differentiated and poorly differentiated neuroendocrine carcinomas^6^. For staging of TETs the Koga modification of the Masaoka Staging system (Masaoka-Koga) is recommended, which classifies TETs according to their level of invasiveness into 4 stages^7^.

TETs and autoimmune diseases: There is only scarce information on the pathophysiology of TETs. Neither risk factors nor biomarkers for diagnosis of TETs have been established^8^. TETs are often associated with a variety of paraneoplastic syndromes, most frequently Myasthenia Gravis (MG)^9^. TETs were detected in approximately 10 to 15% of patients with MG^10^, whereas at least 30% of patients with TETs showed symptoms of associated paraneoplastic MG at time of diagnosis^11^. In contrast, up to 50% of TNETs are associated with paraneoplastic endocrine diseases, most commonly with the hereditary tumor syndrome multiple endocrine neoplasia type 1 (MEN1)-and paraneoplastic Cushing syndrome^12^.

MG and Thymic Abnormalities: MG is an autoimmune disease mediated by autoantibodies directed against acetylcholine receptors (AChR) or other targets at the neuromuscular junction, resulting in characteristic symptoms, like muscle weakness^13^. Up to 80% of patients with MG show thymic abnormalities, comprising TETs^14^ and benign irregularities of the thymus, such as true thymic hyperplasia (TTH) and follicular thymic hyperplasia (FTH). FTH and TTH are clinically summarized as thymic hyperplasia (TH) characterized as macroscopic or radiological enlargement of the thymus, respectively. Microscopically FTH is characterized by the presence of lymphoid follicles with germinal centers (GCs) in the thymic medulla, whereas TTH shows a non-age related, regular lobulated thymic tissue with no or only few signs of fatty involution^15^.

In our previous work we have provided evidence for a role of the receptor for advanced glycation endproducts (RAGE) and its ligand high mobility group box 1 (HMGB1) in MG^16^, TETs, TTH, FTH and regular thymus^17^. HMGB1 is an important extracellular, cytoplasmic and nuclear regulator of apoptosis and autophagy^18^,^19^. Extracellular HMGB1 stimulates autophagy via binding to RAGE, cytoplasmic HMGB1 binds to Beclin1-binding protein and nuclear HMGB1 controls autophagy by its downstream molecule heat shock protein 27 (HSP27/HSPB1)^18^. Vice versa heat shock protein 70 (HSP70) stimulates activation of NFκB and c-Jun terminal kinase (JNK)/Beclin-1 resulting in a positive feedback mechanism leading to increased expression and release of HMGB1^20^. These observations sparked our interest in the possible involvement of HSPs in thymic pathology.

Heat shock proteins (HSPs) are highly conserved proteins that are present in nearly all nucleated cells^21^. HSPs are classified according to their molecular weight: HSP100, HSP90, HSP70, HSP60, HSP40 and small HSPs (15–30kDa) including HSP27^22^. Physiologically HSPs are kept at low levels and are induced by various environmental and pathophysiological stressors, such as heat shock, radiation, hypoxia and reactive oxygen species^23^,^24^. Two main functions of HSPs are known: first HSPs act as molecular chaperones thereby playing a role in intracellular protein folding, aggregation, transport and homeostasis^24^,^25^. Second HSPs have important functions as inhibitors of caspase-dependent (e.g. HSP27, HSP70) and -independent apoptosis pathways (e.g. HSP70)^26^.

HSPs as targets for cancer treatment: Because of their anti-apoptotic functions HSPs are believed to be key players in the pathogenesis of malignancies and as targets for novel cancer treatment strategies^27^. In cell culture based studies HSPs promoted tumorigenesis by increasing cellular migration^28^, differentiation^29^ and drug resistance^30^.

Tumor Microenvironment: Tumor cells are surrounded by a variety of cell types, such as endothelial cells (ECs), fibroblasts and macrophages. Together these cells and the extracellular matrix comprise the tumor microenvironment that contributes one of the hallmarks of cancer, which is essential for tumor promotion and progression^31^,^32^. Recently it was shown that heat shock factor 1 (HSF1), the main transcription factor for HSPs, is a potent enabler of malignancy and high stromal expression in early-stage lung and breast cancer is strongly associated with poor clinical outcome^33^.

In the present study we sought to investigate the role of the strongest inducible heat shock proteins (HSP27 and 70)^21^ in TETs, tumor microenvironment and benign thymic alterations of fetal, infantile and adult patients. Moreover we measured serum concentrations of the indicated HSPs in patients with TETs, TH and volunteers with and without MG.

## Results

### HSP expression in TETs, TNETs, TH and regular thymus

#### Expression of HSP27 and 70 in TETs and TNETs

##### HSP and WHO type

**Figure 1:**
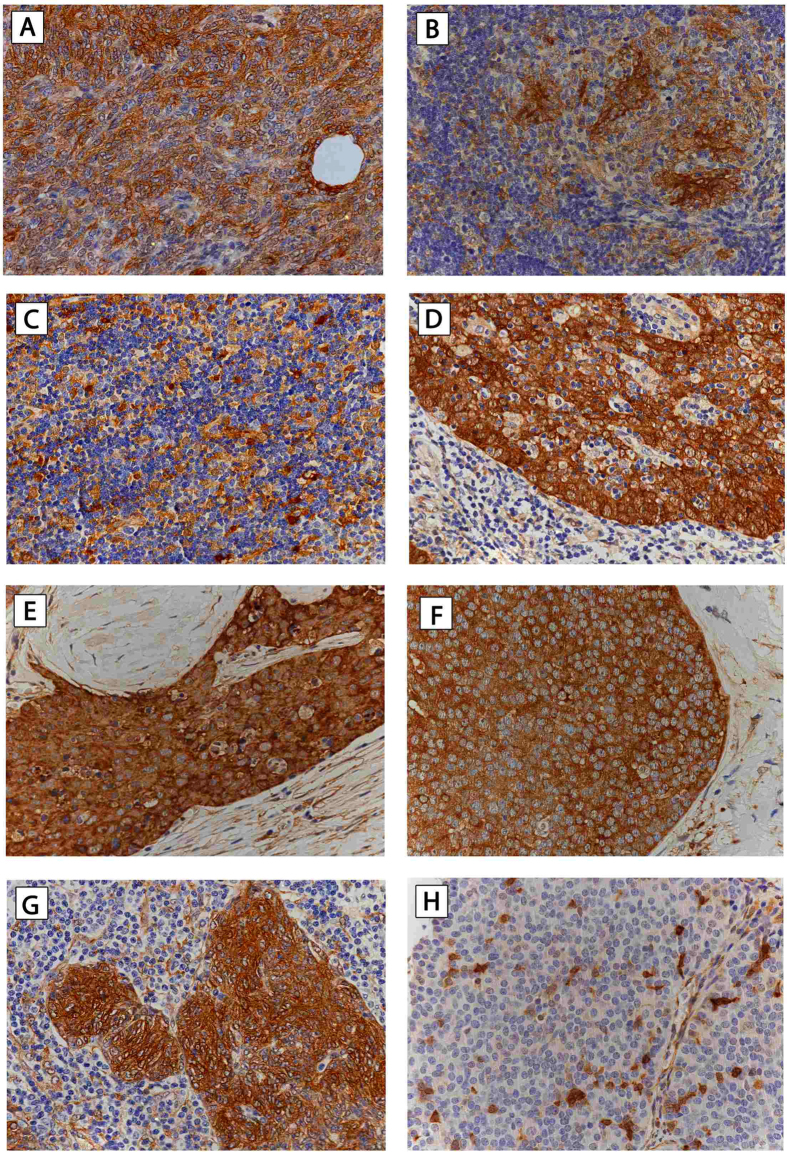
HSP27 expression in TETs. Immunohistochemical staining of HSP27 in different histologic tumor subtypes was performed. Expression of HSP27 in thymoma WHO type A (**A**), AB (**B**), B1 (**C**), B2 (**D**), B3 (**E**), TC-SCC (**F**), MNT (**G**), and TNET (**H**), is shown *(400X magnification).* Hematoxylin was used for counterstaining***. HSP27**,* Heat Shock Protein 27; ***TETs**,* Thymic Epithelial Tumors; ***WHO**,* World Health Organization; ***TC***, Thymic Carcinoma; ***SCC**,* Squamous Cell Carcinoma; ***MNT***, Micronodular Thymoma; ***TNET***, Thymic Neuroendocrine Tumor.

**Figure 2:**
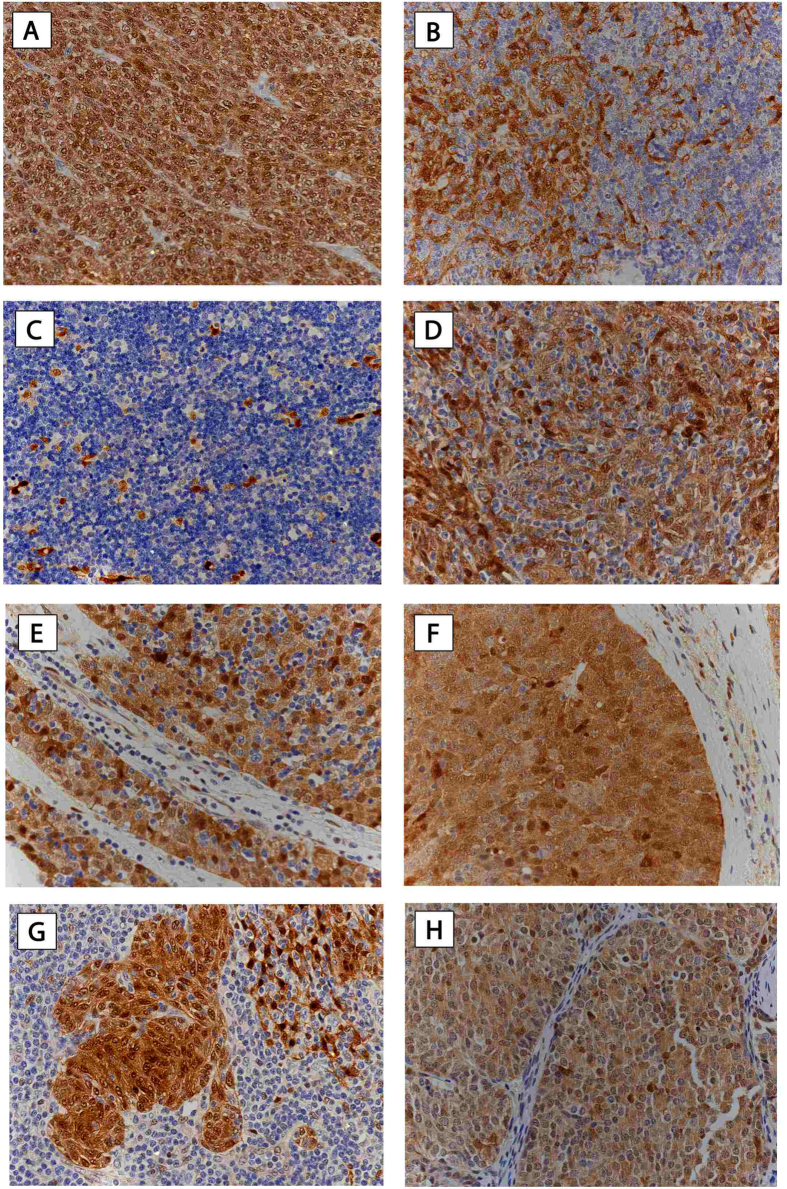
HSP70 expression in TETs. Immunohistochemical staining of HSP70 in different histologic tumor subtypes was performed. Expression of HSP70 in thymoma WHO type A (**A**), AB (**B**), B1 (**C**), B2 (**D**), B3 (**E**), TC-SCC (**F**), MNT (**G**), and TNET (**H**), is shown *(400x magnification).* Hematoxylin was used for counterstaining***. HSP70**,* Heat Shock Protein 70; ***TETs**,* Thymic Epithelial Tumors; ***WHO**,* World Health Organization; ***TC***, Thymic Carcinoma; ***SCC**,* Squamous Cell Carcinoma; ***MNT***, Micronodular Thymoma; ***TNET***, Thymic Neuroendocrine Tumor.

Semiquantitative analysis revealed moderate to strong expression of HSP27 and 70 in all investigated types of thymomas and TCs, in contrast to low or absent expression in well-differentiated TNETs (HSP27: p < 0.001; HSP70 nuclear: p = 0.003, cytoplasmic: p = 0.061; HSP27 was detected in the cytoplasmic but not the nuclear compartment of tumor cells, whereas HSP70 showed nuclear and cytoplasmic expression. HSP70 expression was more intense in nuclei compared to the cytoplasm (nuclear 2.39 ± 0.1 vs. cytoplasmic 2.11 ± 0.1; p = 0.023), (Table 1; Figs 1 and 2; figures with higher magnification are available as supplementary material, Suppl. Figs 1 and 2).

HSP27 expression in tumor cells correlated significantly with WHO classification (p = 0.013; r = 0.247). There was no significant correlation for HSP70 expression with WHO histology (HSP70 nuclear: p = 0.173; r = −0.137, cytoplasmic: p = 0.437; r = −0.078). HSP27 expression in tumor cells correlated statistically significant with nuclear and cytoplasmic HSP70 expression (p = 0.027; r = 0.221 and p = 0.019; r = 0.235).

HSP staining in TETs in comparison to immunohistochemical markers used for routine diagnosis can be found as supplementary material (Suppl. Fig. 3, Suppl. Table 1).

HSP and Masaoka-Koga stage:

There was no significant difference in HSP27 staining intensities in TETs from patients diagnosed at different Masaoka-Koga stages (p = 0.770). Analysis of HSP70 staining intensities in tumor cells of different Masaoka-Koga stages revealed significant differences for nuclear (p = 0.037), but not for cytoplasmic HSP70 expression (p = 0.261). Nuclear HSP70 expression in stage I–III was higher compared to stage IV TETs (p = 0.046), while no difference was observed for cytoplasmic HSP70 expression (p = 0.100). Nuclear HSP70 expression was significantly higher in non-invasive TETs (stage I) compared to invasive TETs (stage II–IV) (p = 0.045; Table 1). There was no statistically significant correlation between HSP27 and 70 staining intensities and Masaoka-Koga stage (HSP27: p = 0.082, r = 0.175; HSP70 nuclear: p = 0.106, r = −0.162; cytoplasmic: p = 0.084, r = −0.173).

##### HSP and MG

We did not detect significant differences in staining intensities of HSP27 (p = 0.058) and HSP70 (nuclear: p = 0.656; and cytoplasmic: p = 0.558, respectively) in TETs of patients with or without MG.

##### HSP expression and recommended outcome measures for TETs

Overall survival (OS) and cancer specific survival (CSS) in TETs with low cytoplasmic HSP27 expression were not significantly different compared to TETs with moderate to strong HSP27 expression (p = 0.867 and p = 0.352, respectively). Freedom from recurrence (FFR) was significantly worse in TETs with low HSP27 expression (p = 0.017).

**Figure 3:**
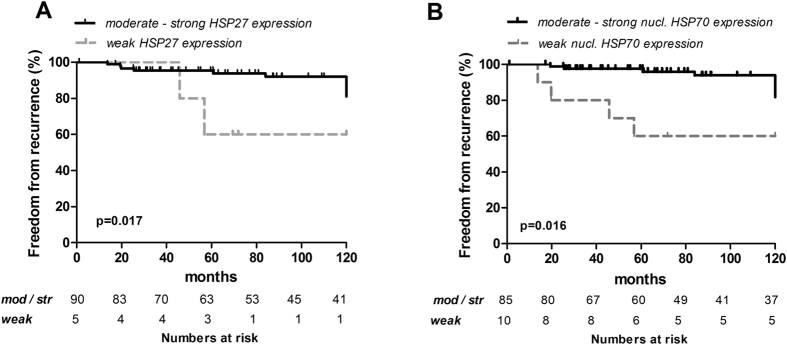
HSP expression and freedom from recurrence in TETs. Kaplan-Meier survival plots for HSP27 (**A**) and HSP70 (**B**) and FFR are shown. The number of patients at risk for moderate to strong and weak HSP27 and 70 expression are listed for the indicated time points. ***HSP27,***Heat Shock Protein 27; ***HSP70**,* Heat Shock Protein 70; ***TETs**,* Thymic Epithelial Tumors; ***FFR,***Freedom From Recurrence.

Cytoplasmic HSP70 staining intensities in TETs showed no statistically significant differences in OS (p = 0.337), CSS (p = 0.291) and FFR (p = 0.082), respectively. Weak nuclear HSP70 expression in TETs was associated with significantly worse FFR compared to TETs with moderate to strong nuclear HSP70 expression (p = 0.016). There was no significant difference of OS and CSS between TETs with weak nuclear HSP70 compared to those with moderate to strong expression (p = 0.980 and p = 0.985, respectively; Table 2; Fig. 3).

#### HSP and tumor microenvironment

##### HSP27 and 70 expression in stromal cells of the tumor microenvironment

HSP27 and 70 were both expressed in ECs, fibroblasts, adipocytes and macrophages but were absent in lymphocytes (HSP27: p < 0.001, cytoplasmic HSP70: p < 0.001). HSP27 showed significantly stronger expression in ECs compared to fibroblasts, adipocytes or macrophages (p < 0.001) and was even stronger than HSP27 expression in TETs (p = 0.035).

The strongest HSP70 expression in cells of the tumor microenvironment was found in macrophages (p < 0.001). Macrophages in the tumor microenvironment of WHO type AB thymomas showed the strongest HSP70 expression which was significantly stronger compared to macrophages of type A thymomas (p = 0.001). The weakest HSP70 expression in fibroblasts was found in WHO type B3 thymomas which was significantly lower compared to fibroblasts of the tumor microenvironment in type AB thymomas (p ≤ 0.001). It is noteworthy that cytoplasmic HSP70 expression in TETs was higher than staining intensities of stromal cells (p < 0.001).

Stromal cells showed significantly stronger expression of HSP27 2.3 ± 0.0 compared to HSP70 1.0 ± 0.0 (p < 0.001). Paraneoplastic MG had no influence on HSP expression in cells of the tumor microenvironment (Suppl. Table 2).

#### Benign Thymic Alterations and Regular Thymus

##### Role of HSPs in regular and hyperplastic thymus

**Figure 4:**
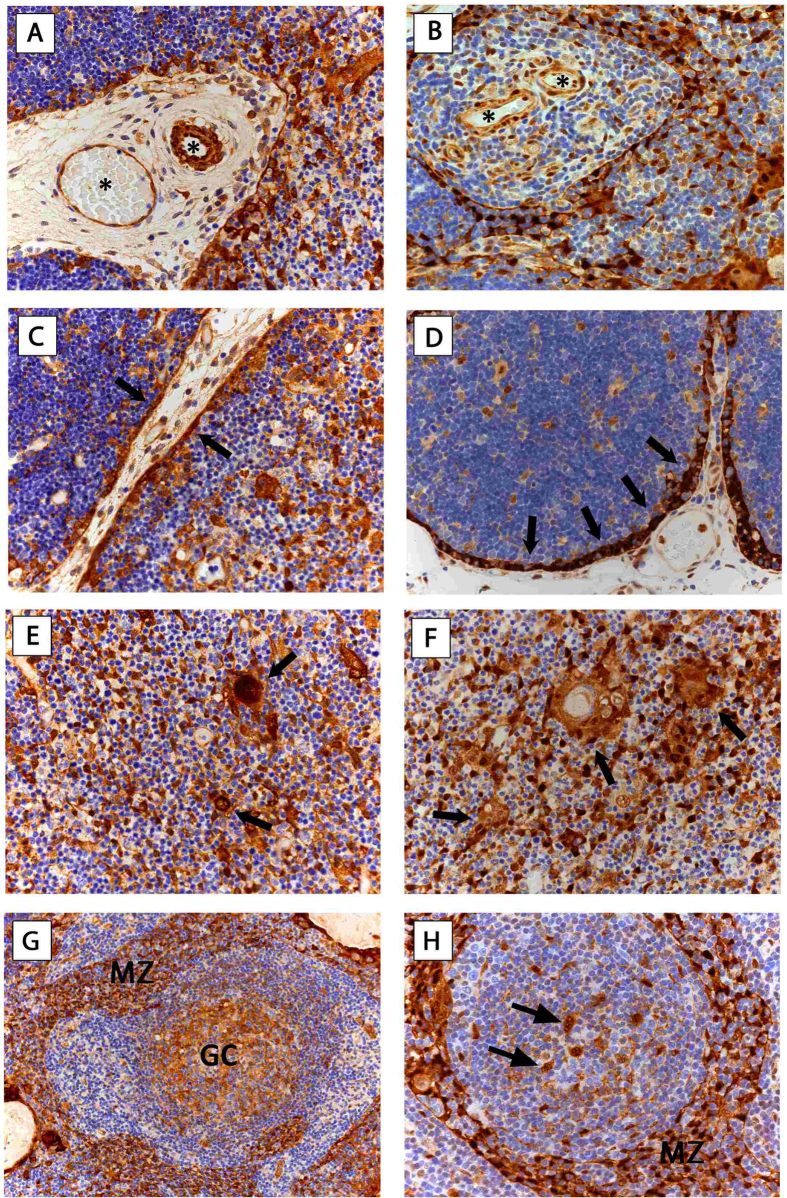
HSP27 and HSP70 expression in non-malignant thymic specimens. Representative images show endothelial expression of HSP27 (**A**) in fetal thymus and HSP70 (**B**) in adult thymus with regular morphology (*asterisk). cTECs with intense subcapsular expression of HSP27 (**C**) in fetal thymus and HSP70 (**D**) in FTH is shown (arrows). mTECs and HCs express HSP27 (**E**) and HSP70 (**F**) in fetal thymus. Arrows indicate HCs. Staining of HSP27 (**G**) and HSP70 (**H**) in dendritic cells of GCs (GC) and TECs in MZ-like distribution (MZ) in FTH is shown. Arrows indicating HSP expressing dendritic cells in GCs *(A-F, H: 400*x *magnification; G: 200*x *magnification).* Hematoxylin was used for counterstaining.***HSP27**,* Heat Shock Protein 27; ***HSP70**,* Heat Shock Protein 70; ***cTEC***, cortical Thymic Epithelial Cells; ***mTEC***, medullary Thymic Epithelial Cells; ***FTH***, Follicular Thymic Hyperplasia; ***HC***, Hassall’s Corpuscles; ***GC**,* Germinal Center; ***MZ**,* TECs with marginal zone like distribution.

In ECs we found strong expression of HSP27 in all specimens (n = 23) and strong expression of HSP70 in 95.6% of cases. Further we detected strong expression of HSP27 and 70 in medullary-(mTECs) and cortical TECs (cTECs) in all non-malignant thymic specimens (n = 24). All cTECs showed strong expression of both HSPs. All Hassall’s corpuscles in the thymic medulla expressed HSP27 and 70 (Fig. 4).

### HSP expression in germinal centers of thymus of MG

Additionally, we found HSP27 and 70 expression in dendritic cells (DCs) of GCs and TECs with mantle zone (MZ)-like distribution in all patients with MG (FTH: n = 6). In accordance to TETs, lymphocytes were negative in all cases of non-neoplastic thymic tissues, in particular those with MG (Fig. 4).

We did not detect statistically significant difference in staining patterns between patients with or without MG, in fetal, infantile and adult thymic specimens, respectively (Suppl. Table 3).

### HSPs-markers for TEC and thymic dendritic cells

**Figure 5:**
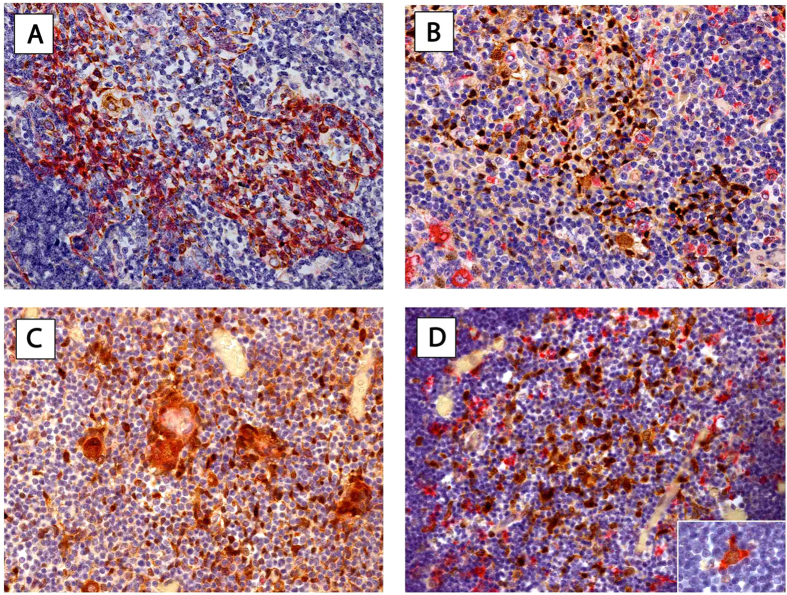
Immunophenotyping of HSP expressing cells in fetal and adult thymus with regular thymic morphology. Double-staining of Lu5-pancytokeratin (red) and HSP70 (brown) of an adult (**A**) and fetal thymus (**C**) is shown. There was complete concordance of Lu5 and HSP70 expression. Staining pattern of CD68 (PGM-1, macrophages/DCs) (red) and HSP70 (brown) double-staining is shown for an adult (**B**) and fetal (**D**) thymus. While most cells showed either distinct CD68 or HSP70 expression we found few cells that showed double staining (insert in D 630x magnification). Hematoxylin was used for counterstaining. *Magnification: 400x. **HSP27***, Heat Shock Protein 27; ***HSP70***, Heat Shock Protein 70; ***DCs***; dendritic cells.

To specify the type of HSP expressing thymic cells we performed sequential double-staining of fetal and adult non-malignant thymic specimens. Lu-5 staining was used as marker for TECs whereas CD68 (PGM-1) was used to identify macrophages and DCs. Double-staining of Lu-5/HSP70 and CD68/HSP70 in adult and fetal thymic specimens was performed all thymic cells that showed positive staining for HSP70 coexpressed Lu5. The majority of thymic cells showed single staining for HSP70 or CD68. Only a subset with simultaneous expression of HSP70 and CD68 was identified (Fig. 5).

### Analysis of HSPs in serum of patients with thymic abnormalities

#### HSP concentrations in TETs

##### Increased HSP27 and 70 serum concentrations in patients with TETs and correlation with histologic tumor subtype

There was no significant statistical difference considering age (p = 0.376) and sex (p = 0.477) in patients with TETs and healthy volunteers.

We found significantly increased serum concentrations of HSP27 and 70 in patients with TETs compared to healthy volunteers (HSP27[pg/ml]: 509.6 ± 67.2 vs. 334.6 ± 50.2; p = 0.040 and HSP70[ng/ml]: 2.0 ± 0.3 vs. 1.3 ± 0.1; p = 0.021, respectively). Further stratification of patients with TETs into patients with TCs and thymomas compared to volunteers revealed significant differences of HSP27 and 70 serum concentrations (HSP27[pg/ml]: 635.9 ± 118.3 vs. 412.5 ± 73.2 vs. 334.6 ± 50.2; p = 0.021 and HSP70[ng/ml]: 2.4 ± 0.5 vs. 1.7 ± 0.3 vs. 1.3 ± 0.1; p = 0.015, respectively). Post hoc comparisons revealed significant differences in HSP27 and 70 serum concentrations only in patients with TCs compared to volunteers (HSP27[pg/ml]: 635.9 ± 118.3 vs. 334.6 ± 50.2; p = 0.017 and HSP70[ng/ml]: 2.4 ± 0.5 vs. 1.3 ± 0.1; p = 0.012, respectively). Separate analysis of HSP27 and 70 serum concentrations in patients with thymomas compared to volunteers showed no significant differences (HSP27[pg/ml]: 412.5 ± 73.2 vs. 334.6 ± 50.2; p = 0.374 and HSP70[ng/ml]: 1.7 ± 0.3 vs. 1.3 ± 0.1; p = 0.161, respectively).

**Figure 6:**
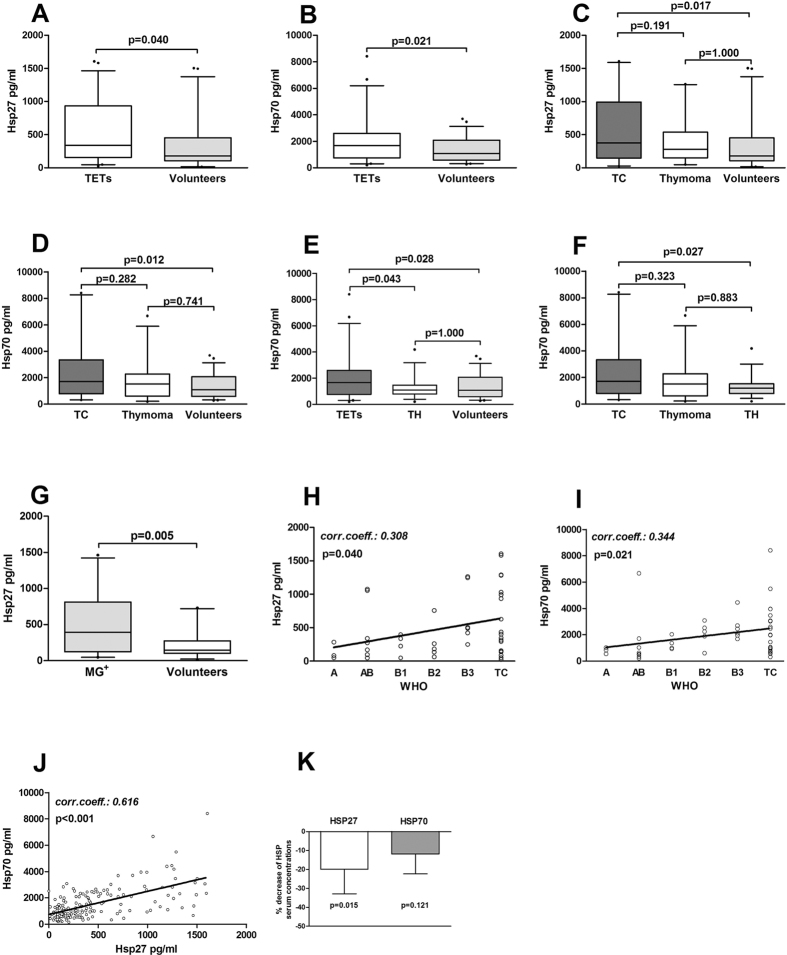
Serum concentrations of HSP27 and 70 in patients with TETs, TH, MG and volunteers. Serum concentrations of HSP27 (**A**) and HSP70 (**B**) in patients with TETs compared to healthy volunteers are shown. Patients with TETs were further separated into patients with TC and thymoma. Serum concentrations for HSP27 (**C**) and HSP70 (**D**) are shown. Serum levels of HSP70 in patients with TETs compared to patients with TH and volunteers is shown (**E**). Analysis of HSP70 serum concentrations in patients with TC compared to thymomas and TH is shown (**F**). Serum concentrations of HSP27 in patients with MG compared to volunteers is shown (**G**). Spearman rho correlations for HSP27 (**H**) and HSP70 (**I**) with WHO classification is shown. Pearson correlation for HSP27 and HSP70 serum concentrations are shown (**J**). Paired T-Test was performed to analyze fold decrease of HSP27 and 70 serum concentrations after tumor resection (**K**). ***HSP27***, Heat Shock Protein 27; ***HSP70***, Heat Shock Protein70; ***TETs***, Thymic Epithelial Tumors; ***TH***, Thymic Hyperplasia; ***MG***, Myasthenia Gravis; ***TC***, Thymic Carcinoma; ***r,***correlation coefficient.

HSP27 and 70 serum concentrations correlated significantly with WHO tumor classification (HSP27: p = 0.040; r = 0.308 and HSP70: p = 0.021; r = 0.344). In particular, the lowest HSP serum concentrations were found in WHO type A and AB thymomas, whereas the highest concentrations were found in B3 thymomas and TCs (Table 3; Suppl. Table 4; Fig. 6).

### Increased HSP serum concentrations in advanced Masaoka-Koga tumor stages

Patients with Masaoka-Koga stage III–IV TETs showed significantly higher HSP27 serum concentrations compared to stage I–II tumors (HSP27[pg/ml]: 647.8 ± 100.4 vs. 358.9 ± 78.4; p = 0.030). There was no statistically significant difference in HSP70 serum concentrations when stages I-II vs. III-IV were analyzed (HSP70[ng/ml]: 1.6 ± 0.3 vs. 2.4 ± 0.4; p = 0.111). Serum HSP concentrations of non-invasive (Masaoka-Koga I) and invasive TETs (Masaoka-Koga II–IV) were not significantly different (HSP27[pg/ml]: 446.2 ± 198.7 vs. 521.0 ± 71.9; p = 0.789 and HSP70[ng/ml]: 1.4 ± 0.4 vs. 2.1 ± 0.3; p = 0.203; Table 3).

We did not find significant correlations between Masaoka-Koga stage and HSP27 (p = 0.098; r = 0.248) or HSP70 (p = 0.084; r = 0.258), respectively.

#### Decreased serum concentrations of HSPs after surgical tumor resection

Longitudinal analysis revealed significantly decreased HSP27 serum concentrations after complete tumor resection (0.199 fold decrease, p = 0.015). Although we detected also a decrease of HSP70 serum concentrations after tumor resection, the difference was not statistically significant (0.118 fold decrease, p = 0.121; Table 3; Fig. 6K).

#### Comparison of HSP concentrations in TETs and TH

##### Differences in HSP serum concentrations in patients with TETs compared to TH

Analysis of HSP27 serum concentrations in patients with TETs compared to TH and volunteers showed no significant differences (HSP27[pg/ml]: TETs 509.6 ± 67.2 vs. TH 464.6 ± 80.1 vs. volunteers 334.6 ± 50.2; p = 0.115). In contrast HSP70 serum concentrations were significantly increased in patients with TETs compared to both volunteers (p = 0.028) and patients with TH (p = 0.043; HSP70[ng/ml]: TETs 2.0 ± 0.3 vs. TH 1.3 ± 0.1 vs. volunteers 1.3 ± 0.1). There was no significant difference between HSP70 serum concentrations in TH and volunteers (p = 1.000).

Separate analysis of HSP27 and 70 serum concentrations in patients with TCs, thymomas and TH revealed significantly increased HSP70 serum concentrations only in TCs compared to TH (p = 0.027), whereas there was no significant difference for HSP27 serum concentrations between those subgroups (p = 0.236; HSP27[pg/ml]: TC 635.9 ± 118.3 vs. thymoma 412.5 ± 73.2 vs. TH 464.6 ± 80.1 and HSP70[ng/ml]: TC 2.4 ± 0.5 vs. thymoma 1.7 ± 0.3 vs. TH 1.3 ± 0.1; Table 3; Fig. 6).

#### HSP concentrations in MG

##### Higher HSP serum concentrations in patients with autoimmune MG

There was no significant difference in HSP27 and 70 serum concentrations in patients with TETs with or without paraneoplastic MG (HSP27[pg/ml]: TETs MG^+^ 434.6 ± 120.2 vs. TETs MG^−^ 533.2 ± 80.4; p = 0.538 and HSP70[ng/ml]: TETs MG^+^ 1.9 ± 0.3 vs. TETs MG^−^ 2.0 ± 0.3; p = 0.836; Table 3).

Emphasizing on MG, we measured HSP27 and 70 serum concentrations in a subgroup of 26 patients with MG (11 TETs, 15TH) compared to 26 sex (p = 0.760) and age (p = 0.453) matched volunteers. We detected significant higher serum concentrations of HSP27 in MG patients compared to volunteers (HSP27[pg/ml]: 507.5 ± 89.5 vs. 215.5 ± 35.9; p = 0.005), whereas there was no significant difference for HSP70 (HSP70[ng/ml]: 1.5 ± 0.2 vs. 1.1 ± 0.2; p = 0.088; Table 3; Fig. 6).

## Discussion

This is the first description of HSP27 and 70 expression in human regular thymic morphology of different developmental stages (fetuses, infants and adults), in non-neoplastic thymic diseases, namely TTH and FTH and neoplasms of thymic epithelial origin.

HSPs are overexpressed in several malignancies, including colon cancer^34^, prostate cancer^35^ hepatocellular carcinoma (HCC)^36^, lung cancer^37^ and oral squamous cell carcinoma (SCC)^38^. HSP overexpression in cancer is related to tumor growth, resistance to chemotherapy, metastases and poor survival^39^,^40^. Overexpression of HSPs was associated with inhibition of apoptosis and favored tumor initiation and progression.These observations may also be of relevance in regard to our data on thymomas and TCs. Semiquantitative analysis of HSP 27 and 70 revealed high staining intensities in all WHO type thymomas, MNT and TCs. Nuclear and cytoplasmic HSP70 expression gradually decreased from Masaoka-Koga tumor stage I to IV, indicating that a decrease of HSP70 expression goes along with increasing malignant tumor invasiveness and worse prognosis. Higher expression of HSPs in TETs of lower Masaoka-Koga stages is in line with SCC of the tongue where overexpression of HSP27 is associated with early tumor stages and grade 1 tumors^38^ and Non-Small Cell Lung Cancer (NSCLC) where overexpression of HSP27 and 70i were favorable prognostic factors for survival^37^. These observations are in contrast to findings of overexpression in carcinomas of other sites with worse prognosis: HSP70 overexpression in colon cancer with low tumor differentiation and advanced tumor stage^34^, strong expression of HSP27 and 60 in prostate cancer with shorter recurrence free survival^35^, or in HCC where high HSP70 expression correlated with higher tumor stage, greater tumor size and invasiveness^36^.

In our patient cohort: 1-, 5- and 10-year OS (and CSS) after primary tumor resection were 100% (100%), 96.5% (97.8%) and 90.9% (94.3%). Because of this excellent survival after complete resection with or without multimodality treatment in cases of advanced tumor stage^41^ and the fact that recurrences can occur even decades after primary tumor resection [Cumulative Incidence Rates (CIR) of 0.12 are reported^42^]-the biology of TETs is best represented by FFR^43^. We showed that 10-year FFR was significantly worse in TETs with weak HSP27 and 70 expression (87.5% and 60.0%, respectively) compared to TETs with stronger HSP27 and 70 expression (91.4% and 93.9%, respectively).

In contrast to gradually decreasing nuclear and cytoplasmic HSP70 expression in TETs, serum concentrations of HSP70 incrementally increased from Masaoka-Koga tumor stage I to IV. Similar observations were reported in patients with NSCLC with higher serum concentrations of HSP27 in stages IIIA-IV compared to stages I-II^44^.

Since there is no information on the expression of HSPs in TNETs in the peer reviewed literature we compare our results to HSPs in neuroendocrine tumors (NETs) of other origins. HSP90 was reported to be highly expressed for instance in pancreatic^45^ and pulmonary neuroendocrine tumors^46^. In stark contrast to the high HSP90 expression in NETs of other origins we found significantly lower expression of HSP27 and 70 in TNETs. We propose the following reasons for this discrepancy: TNETs in comparison to thymomas and NETs of other origins show a more aggressive behavior^47^ with 5-year CSS of 50% for well-differentiated, 20% for moderately differentiated and 0% for poorly differentiated TNETs^48^. The majority of TNETs present in advanced tumor stages (Masaoka-Koga Stage III-IV). TNETs showed significantly higher 5- and 10-year CIR (0.34 and 0.54, respectively) compared to thymomas^12^, confirming the more aggressive clinical behavior of these neoplasms.

Tumor-promoting inflammation was described as an enabling characteristic fostering multiple hallmarks of cancer: independence from growth factors, escape from programmed cell death, enhanced angiogenesis and metastasis^32^. Extracellular HSP27 and 70 act as danger signals providing a chronic inflammatory tumor environment that promotes tumor progression and invasion^49^. We found elevated serum concentrations of HSP 27 and 70 in patients with TETs and strong expression of HSP27 and 70 in macrophages of the tumor microenvironment contrasting the finding of only few CD68/HSP70 double positive cells in fetal and adult non-neoplastic specimens. Longitudinal measurements showed that HSP27 serum concentrations were significantly decreased after complete surgical resection of TETs. This effect could be either related to direct release from tumor cells or indirect release from immune cells within the framework of cancer-related inflammation. Similar to our findings significantly decreased HSP27 concentrations were found after surgical resection of lung metastases in patients with metastatic colorectal cancer (CRC)^50^. HSP27 as a tumor marker is promising and warrants further studies.

Thymocytes that pass positive and negative selection are mature T-cells that will be released from the thymus. During this maturation process thymocytes migrate through the epithelial framework from thymic cortex to medulla^51^. Double-staining of fetal and adult regular thymic specimens showed that HSPs are constitutively expressed in TECs. The role of HSP27 and 70 in mTECs and cTECs, which form the three-dimensional framework in the thymus that enables T-cell maturation and development warrants future study that may improve our understanding of the basic mechanisms of the thymic microenvironment and T-cell maturation.

Involvement of HSPs in the pathogenesis of several autoimmune diseases, such as Systemic Lupus Erythematosus^52^, diabetes^53^ and multiple sclerosis^54^ were reported. Elevated levels of serum HSP70-antibodies could be found in patients with generalized and ocular MG^55^. Our finding of HSP27 and 70 expressing DCs and TECs forming marginal zone-like distributions in the GCs of thymus may add to the intrathymic pathogenesis of MG. Briefly, nicotinic AChRα epitopes are presented to autoreactive CD4^+^ thymic immigrants by thymic antigen presenting (possibly HSP expressing) DCs. This may lead to their activation and help in the stimulation of AChR-reactive B cells forming GCs that produce anti-AChR antibodies that is one of the hallmarks of MG development^56^.

Currently several HSP90 inhibitors are under investigation in preclinical and clinical phase I to III trials^57^. Promising results were reported while the exact mechanisms of their anti-cancer effects remain largely unknown. Especially the approach to use HSP90 inhibitors combined with other antineoplastic drugs has shown promising results^58^. Monotherapy with HSP inhibitors showed considerable adverse drug effects^57^. The observed toxicity of HSP inhibition may result from targeting the constitutive expression of HSP in rest of the body (investigated in our study the high expression in TECs, ECs, macrophages, fibroblasts and adipocytes). Combination of HSP inhibitors can reduce drug resistance and toxicity of chemotherapy^57^.

### Limitations of the study

This study carries the weaknesses of other important studies done in orphan diseases^59^. The rarity of TETs and their vast array of different histological patterns, different tumor stages and association with autoimmune diseases does not allow for further significant categorization of patients. This is particularly limiting for studies on patient serum that are widely not routinely sampled. Orphan disease research on TETs and MG needs international efforts such as those of the European Society of Thoracic Surgeons (ESTS) Thymic Workgroup and the International Thymic Malignancy Interest Group (ITMIG)^42^,^43^. In order to maximize the number of available tumor tissue samples and to allow for long follow-up periods this study was carried out in a prospective and retrospective manner (median follow-up time for the analysis of tumor tissue analysis: 111.7 months). Because of excellent survival and late recurrences-up to 20 years in patients with TETs^60^-the reported follow-up periods are relatively short. The number of experimentally investigated TNETs is particularly small. A recent multi-institutional outcome study on TNETs (not experimental research on tumor tissue or serum) reported on 205 patients^12^.

### Summary

In summary, we showed for the first time that HSP27 and 70 are expressed in TETs, the thymic tumor microenvironment and regular thymus. Elevated serum concentrations were detected in patients with TETs and correlated with advanced tumor stages. Longitudinal analysis identified HSP27 serum concentration as a promising tumor marker. Altogether HSP27 and 70 may have diagnostic implications and may serve as molecular targets for therapy. Further studies are warranted for a more detailed understanding of HSPs in thymic malignancies, MG and thymic physiology.

## Materials and Methods

### Study design and study population

We included 125 patients in this study who underwent thymectomy at the Department of Thoracic Surgery/Medical University Vienna between 1999 and 2014. Indications for surgery can be summarized as follows: 1) histologically verified TET, 2) radiological suspicion of TET (mediastinal mass) or 3) thymectomy in patients with MG.

According to histology specimens were classified as thymic malignancies or non-malignant thymic specimens (n = 24). We analyzed tissue specimens of 101 patients with TETs (thymoma: n = 83; TC: n = 15; TNET: n = 3) who underwent surgical resection. Mean age at surgery was 57.8 years (range 16.7–86.5). Sixty-one female (60.4%) and 40 male (39.6%) patients were included. TETs were further classified according to WHO classification system^3^. For the purpose of statistical analysis of this manuscript WHO mixed type thymomas were classified according to the more malignant part (e.g. B2/B3 thymoma was classified as B3 thymoma).

Non-malignant thymic specimens (n = 24) were distinguished between regular for age thymic morphology (n = 8), TTH (n = 10) and FTH (n = 6). Fetal and infantile thymic tissues for histological processing were obtained at autopsy.

Serum samples of 46 patients with TETs (11 MG pos., 35 MG neg.), 33 patients with TH (15 MG pos., 18 MG neg.) and 49 volunteers were available. For a subgroup of 16 patients with TETs pre- and postoperative serum samples were available (mean 13.94 months after surgery). For repeated measures patients had to fit to the following criteria: complete surgical resection of TET (R0), no adjuvant therapy during the last month, and no sign of recurrence or 2^nd^ malignancy.

Ethical approval was obtained from the ethics committee of the Medical University Vienna and was conducted according to Good Scientific Practice. Written informed consent was obtained from all participating patients and volunteers and all experiments were carried out in accordance to the approved ethical guidelines.

### Immunohistochemistry

Formaldehyde-fixed and paraffin embedded human benign and malignant thymic specimens were retrieved from the institutional department of pathology. Immunohistochemistry staining was performed by using the automated Ventana Benchmark^®^ platform (Ventana Medical Systems, Tucson, AZ, USA) according to standard protocol of the institutional department of pathology^17^. Monoclonal mouse anti-human HSP27 IgG_2a_ (Santa Cruz, Dallas, Texas, USA), monoclonal mouse anti-human HSP70 IgG_2a_ (Novus, Littleton, CO, USA) and anti-mouse IgG secondary antibodies (Vector Laboratories, Burlingame, CA, USA) were used for staining. Omission of primary antibody served as negative control. Further information to antibody specificity of HSP27 and 70 are available as supplementary material.

### Double-Staining

Sequential double-staining on formaldehyde-fixed and paraffin embedded human non-malignant thymic specimens was performed by using the automated Leica Bond III immunostainer platform (Leica Biosystems, Nussloch, Germany). The following antibodies were used: CD68 (clone PGM-1) mouse monoclonal antibody IgG3 (DAKO M876, Glostrup, Denmark), Lu-5 (pan cytokeratin) mouse monoclonal antibody IgG1 (Biocare Medical CM43C, Concord, CA, USA) and HSP70/HSPA1A mouse monoclonal antibody IgG_2a_ (Novus Biological, NB600-571, Littleton, CO, USA).

### Evaluation of immunoreactivity

#### Thymic Epithelial Tumors

For the evaluation of immunoreactivity we used a scoring system from 0 to 3 to assess staining intensity of HSP27 and 70 in TETs (0, no staining, 1, weak staining, 2, moderate staining, 3 strong staining)^17^. Analysis of immunoreactivity was performed by two observers blinded to Masaoka-Koga Tumor stage, presence of paraneoplastic MG and WHO type. Rating represents the overall impression/average of staining intensity of most of the tumor cells. In case that two ratings differed, the slide was reevaluated and discussed.

#### Non-Malignant Thymic Specimens

For the analysis of immunoreactivity in non-malignant thymic specimens a quantitative score was used (“+”, positive, “−”, negative). Briefly, slides were screened at low magnification and cell types represented in non-malignant thymic tissues, such as ECs, Hassal’s corpuscles, lymphocytes, GCs, cortical, subcapsular and medullary TECs were evaluated.

### Enzyme-linked immunosorbent assay (ELISA)

HSP27 and 70 concentrations in serum samples were measured by using commercially available human HSP27 (R&D Systems, Minneapolis, MN, USA, DuoSet^®^ Human HSP27, DY1580) and HSP70 ELISA kits (R&D Systems, Minneapolis, MN, USA, DuoSet^®^IC Total HSP70/HSPA1A, DYC1663). All ELISA tests were performed according to the Manufacturer’s instructions. Samples were measured in duplicates. Researchers performing the assays and data analyses were blinded to the groups associated with each sample.

### Statistical methods

Statistical analysis of data was performed using SPSS software (version 21; IBM SPSS Inc., IL, USA). Mann-Whitney-U test and Kruskal-Wallis rank test were used to evaluate non-normal distributions with two or more than two groups, respectively. Unpaired student’s *t* test and one-way ANOVA were used to compare means of two or more than two independent groups, for normal (Gaussian) distributions, respectively. *Post hoc* comparisons were computed with Tukey’s-B and Bonferroni correction. Paired student’s *t*-test was used for analyzing two dependent groups. The type of test used is indicated in the table and/or the result section. All data were reported as mean ± standard error of the mean (SEM) in the text of the results section and more detailed as mean (median) ± standard deviation (SEM) in tables. Chi-square test was used to compare binominal variables. The probability of making a type I error was set at α value of 0.05. The null hypothesis was rejected if the *p*-value was less than α. Two-tailed p-values were employed. Pearson correlation (r) was performed to analysis linear relationships between two numerical measurements. Spearman rho correlation (r) was used in case that one or both variables were ranked. Kaplan-Meier survival analysis and log-rank test were used to assess outcome measures for TETs, such as OS, CSS and FFR. GraphPad Prism6 (GraphPad Software Inc., California, USA) was used for graphical display of all box plots, Kaplan-Meier curves and correlations in this manuscript.

## Additional Information

**How to cite this article**: Janik, S. *et al.* HSP27 and 70 expression in thymic epithelial tumors and benign thymic alterations: diagnostic, prognostic and physiologic implications. *Sci. Rep.* **6**, 24267; doi: 10.1038/srep24267 (2016).

## Supplementary Material

Supplementary Information

## Figures and Tables

**Table 1:** HSP27 and 70 expression in TETs and analysis of clinicopathologic characteristics.

Clinicopathologic characteristics		HSP expression in TETs					
		*Cyto HSP27*		*Cyto HSP70*		*Nucl HSP70*	
		*n*	*mean *±* SEM*	*n*	*mean *±* SEM*	*n*	*mean *±* SEM*
**WHO**	A	16	2.63 ± 0.1	16	2.0 ± 0.2	16	2.44 ± 0.2
	AB	14	2.54 ± 0.1	14	2.5 ± 0.2	14	2.61 ± 0.2
	B1	10	2.30 ± 0.2	11	1.55 ± 0.4	11	1.86 ± 0.3
	B2	21	2.79 ± 0.1	21	2.29 ± 0.2	21	2.60 ± 0.2
	B3	17	2.85 ± 0.1	17	2.0 ± 0.1	17	2.38 ± 0.2
	TC	15	2.93 ± 0.1	15	2.1 ± 0.2	15	2.23 ± 0.3
	MNT	4	3.0 ± 0.0	4	2.5 ± 0.3	4	2.5 ± 0.3
	TNET	3	0.5 ± 0.3	3	1.33 ± 0.4	3	0.33 ± 0.3
*p-value*			< 0.001^a^		0.061^a^		0.003^a^
**Masaoka-Koga Stage**	I	23	2.52 ± 0.1	23	2.30 ± 0.1	23	2.59 ± 0.1
	II	46	2.64 ± 0.1	47	2.17 ± 0.1	47	2.39 ± 0.1
	III	15	2.67 ± 0.2	15	1.97 ± 0.2	15	2.37 ± 0.3
	IV	16	2.63 ± 0.2	16	1.78 ± 0.3	16	1.75 ± 0.3
*p-value*			0.770^a^		0.261^a^		0.037^a^

HSP27 and 70 were strongly expressed in all thymomas and TCs but weak or absent in TNETs. Strongest nuclear HSP70 expression was found in non-invasive TETs (Masaoka-Koga stage I), whereas the weakest expression was found in metastatic TETs (Masaoka-Koga stage IV). Paraneoplastic MG had no significant difference on HSP27 and 70 staining intensities in TETs. ***HSP27***, Heat Shock Protein 27; ***HSP70***, Heat Shock Protein 70; ***n***, number; ***Cyto***, cytoplasmic expression; ***Nucl***., nuclear expression; ***WHO***, World Health Organization; ***TC***, Thymic Carcinoma; ***MNT***, Micronodular Thymoma; ***TNET***, Thymic Neuroendocrine Tumor; ***MG***, Myasthenia Gravis; ***SEM***, standard error of the mean.

^a^One-way Anova.

**Table 2:** HSP expression in TETs and ITMIG recommended outcome measures.

ITMIG recommendedoutcome measures	HSP expression in TETs					
	*Cytoplasmic HSP27*		*Cytoplasmic HSP70*		*Nuclear HSP70*	
Overall Survival (OS)	*weak(n = 5)*	*mod/str(n = 90)*	*weak(n = 15)*	*mod/str(n = 80)*	*weak(n = 10)*	*mod/str(n = 85)*
1 year	100%	100%	100%	100%	100%	100%
5 year	100%	96.3%	93.3%	97.2%	100%	96.1%
10 year	66.7%	92%	81.7%	92.4%	87.5%	91.4%
**p-value**^a^	0.867		0.377		0.980	
**Cancer SpecificSurvival (CSS)**	*weak*	*mod/str*	*weak*	*mod/str*	*weak*	*mod/str*
1 year	100%	100%	100%	100%	100%	100%
5 year	100%	97.7%	93.3%	98.7%	100%	97.6%
10 year	66.7%	95.6%	81.7%	96.4%	87.5%	95.3%
**p-value**^a^	0.352		0.291		0.985	
**Freedom FromRecurrence (FFR)**	*weak*	*mod/str*	*weak*	*mod/str*	*weak*	*mod/str*
1 year	100%	100%	100%	100%	100%	100%
5 year	60%	95.3%	72%	97.1%	60%	97.5%
10 year	60%	92%	72%	93.3%	60%	93.9%
**p-value**^a^	0.017		0.082		0.016	

TETs with absent or weak cytoplasmic HSP27 and nuclear HSP70 staining showed significantly worse FFR. There was no significant difference for OS and CSS. ***ITMIG***, International Thymic Malignancy Interest Group; ***HSP***, Heat Shock Protein; ***TETs***, Thymic Epithelial Tumors; ***OS***, Overall Survival; ***CSS***, Cancer Specific Survival; ***FFR***, Freedom From Recurrence; ***n,***number; ***mod/str,*** moderate to strong HSP expression.

^a^Log-rank test.

**Table 3:** Serum concentrations of HSP27 and 70 in TETs, TH and MG.

Groups	n	HSP serum concentrations			
		HSP27 [pg/ml]	*p-value*	HSP70 [ng/ml]	*p-value*
TETs	46	509.6(339.2) ± 456.1(67.2)	0.040^b^	2.0(1.6) ± 1.7(0.3)	0.021^b^
Volunteers	49	334.6(180.5) ± 351.7(50.2)		1.3(1.1) ± 0.9(0.1)	
TC	20	635.9(419.3) ± 528.8(118.3)	0.021^a^	2.4(2.0) ± 2.0(0.5)	0.015^a^
Thymoma	26	412.5(279.8) ± 373.2(73.2)		1.7(1.5) ± 1.4(0.3)	
Volunteers	49	334.6(180.5) ± 351.7(50.2)		1.3(1.1) ± 0.9(0.1)	
TC	20	635.9(419.3) ± 528.8(118.3)	0.027^b^	2.4(2.0) ± 2.0(0.5)	0.034^b^
Volunteers	49	334.6(180.5) ± 351.7(50.2)		1.3(1.1) ± 0.9(0.1)	
Thymoma	26	412.5(279.8) ± 373.2(73.2)	0.374^b^	1.7(1.5) ± 1.4(0.3)	0.161^b^
Volunteers	49	334.6(180.5) ± 351.7(50.2)		1.3(1.1) ± 0.9(0.1)	
TETs	46	509.6(339.2) ± 456.1(67.2)	0.115^a^	2.0(1.6) ± 1.7(0.3)	0.014^a^
TH	33	464.6(312.8) ± 351.7(50.2)		1.3(1.1) ± 0.8(0.1)	
Volunteers	49	334.6(180.5) ± 351.7(50.2)		1.3(1.1) ± 0.9(0.1)	
TC	20	635.9(419.3) ± 528.8(118.3)	0.236^a^	2.4(2.0) ± 2.0(0.5)	0.032^a^
Thymoma	26	412.5(279.8) ± 373.2(73.2)		1.7(1.5) ± 1.4(0.3)	
TH	33	464.6(312.8) ± 351.7(50.2)		1.3(1.1) ± 0.8(0.1)	
TETs MG^−^	35	533.2(337.5) ± 475.5(80.4)	0.538^b^	2.0(1.4) ± 1.9(0.3)	0.836^b^
TETs MG^+^	11	434.6(396.7) ± 398.8(120.2)		1.9(1.9) ± 1.1(0.3)	
MG (TETs + TH)	26	507.5(392.5) ± 456.5(89.5)	0.005^b^	1.5(1.4) ± 0.9(0.2)	0.088^b^
Volunteers*	26	215.5(144.5) ± 182.8(35.9)		1.1(0.8) ± 0.8(0.2)	
Masaoka-KogaStage			0.153^a^		0.472^a^
I	7	446.2(285.0) ± 525.8(198.7)		1.4(1.0) ± 1.0(0.4)	
II	15	318.2(260.2) ± 280.9(72.5)		1.6(1.0) ± 1.6(0.4)	
III	6	694.9(685.1) ± 452.4(184.7)		2.3(1.7) ± 2.0(0.8)	
IV	18	638.3(412.0) ± 508.5(119.9)		2.4(2.0) ± 1.9(0.4)	

Characterization and categorization of patients with TETs according to type of TET, tumor invasiveness and presence of MG. HSP27 and 70 serum concentrations were elevated in patients with TETs, particularly in patients with TCs. There was no significant difference between TETs with or without paraneoplastic MG. In contrast separate analysis of patients with MG (70% thymomatous MG) showed significantly higher HSP27 and 70 serum concentrations compared to volunteers. ***n***, number; ***HSP27***, Heat Shock Protein 27; ***HSP70***, Heat Shock Protein 70; ***TETs***, Thymic Epithelial Tumors; ***TC***; Thymic Carcinoma; ***TH***, Thymic Hyperplasia; ***MG***, Myasthenia Gravis.

^a^One-way ANOVA.

^b^Independent-samples t-test.

^*^Subgroup of 26 patients with MG was sex- and age-matched for separate analysis. ***mean***(***median***)*** *****±***** SD***(***SEM***).
