# Supplementary material for: HSP27 and 70 expression in thymic epithelial tumors and benign thymic alterations: diagnostic, prognostic and physiologic implications
Source: Sci Rep. 2016 Apr 21;6:24267. doi: 10.1038/srep24267 (PMC4838882; doi:10.1038/srep24267)
Supplement: Supplementary Information [file srep24267-s1.pdf]

Supplementary Material

HSP27 and 70 expression in thymic epithelial tumors and benign thymic alterations: diagnostic, prognostic and physiologic implications

S. Janik[1,2], A. I. Schiefer[3], C. Bekos[1,2], P. Hacker[1,2], T. Haider[2,4], W. Klepetko[1], L. Müllauer[3], H. J. Ankersmit[1,2], B. Moser[1*]

[1]Department of Thoracic Surgery, Division of Surgery, Medical University Vienna, Austria

[2]Christian Doppler Laboratory for the Diagnosis and Regeneration of Cardiac and Thoracic Diseases, Medical University Vienna, Austria

[3]Clinical Institute of Pathology, Medical University Vienna, Austria

[4]University Clinic for Trauma Surgery, Medical University Vienna, Austria

[*]corresponding author

Bernhard Moser, MD, PD, Assoc. Prof., FEBTS

Department of Thoracic Surgery, Division of Surgery, Medical University Vienna

Währinger Gürtel 18-20, 1090 Vienna, Austria

FAX: +43 1 40400 69770

Tel: +43 1 40400 67770

E-mail: bernhard.moser@meduniwien.ac.at

Supplementary Table 1

		Immunohistochemical markers									
**WHO**	**Cases n(F:M)**	**HSP27** ***+/-/n.p.***	**HSP70** ***+/-/n.p.***	**Lu 5** ***+/-/n.p.***	**CD5** ***+/-/n.p.***	**CD20** ***+/-/n.p.***	**NCAM** ***+/-/n.p.***	**c-Kit** ***+/-/n.p.***	**Chromo** ***+/-/n.p.***	**Pax 8** ***+/-/n.p.***	**CK5/6** ***+/-/n.p.***
**A**	12 (8:4)	12/0/0	11/0/0*	12/0/0	0/10/2	3/2/7	0/1/11	0/8/4	0/1/11	0/0/12	1/0/11
**AB**	11 (9:2)	11/0/0	11/0/0	10/0/1	1/8/2	4/7/0	0/1/10	1/9/1	0/0/11	0/1/10	0/0/11
**B1**	11 (7:4)	11/0/0	11/0/0	11/0/0	0/8/3	0/10/1	0/1/10	0/7/4	0/0/11	0/0/11	2/0/9
**B2**	17 (7:10)	17/0/0	17/0/0	14/0/3	1/12/4	3/11/3	0/1/16	2/7/8	0/0/17	1/0/16	2/0/15
**B3**	13 (7:6)	13/0/0	13/0/0	12/0/1	0/11/2	0/7/6	1/1/11	0/10/3	1/1/11	0/0/13	1/0/12
**TC**	15 (5:10)	15/0/0	15/0/0	12/0/3	10/5/0	0/7/8	1/9/5	11/2/2	0/5/10	0/1/14	8/1/6
**MNT**	4 (2:2)	4/0/0	4/0/0	4/0/0	0/4/0	0/4/0	0/2/2	0/4/0	0/1/3	0/0/4	0/0/4
**TNET**	3 (2:1)	1/2/0	2/1/0	2/0/1	0/3/0	0/1/2	2/0/1	1/0/2	3/0/0	0/0/3	0/1/2
**Total Number**	**86 (47:39)**	**84/2/0**	**84/1/0***	**77/0/9**	**12/61/13**	**10/49/27**	**4/16/66**	**15/47/24**	**4/8/74**	**1/2/83**	**14/2/70**

Suppl. Table 1. HSP staining and immunohistochemical markers that have been used during routine diagnostic workup of TETs and TNETs. HSP27 and 70 were expressed in 100% of thymomas and TCs, respectively. The epithelial marker pancytokeratin Lu5 was expressed in 100% of TETs thus correlated with 100% expression of HSP27 and 70 in thymomas and TCs ($p<0.001$; $r=1.0$). Immunohistochemical markers such as CD5, CD20, NCAM, c-Kit, Chromogranin A, Pax 8 and CK5/6 were only individually performed for diagnostic purposes and were found as follows: 17.1%, 20.8%, 11.1%, 23.0%, 11.1%, 33.3% and 93.3% of cases, respectively.

In well-differentiated TNETs (n=3) HSP27 and 70 were expressed in 33.3% and 66.6%, respectively. Similar to Lu5 expression in thymomas and TCs, Lu5 was expressed in 100% of TNETs. The expression of CD5, CD20, Pax8, CK5/6 c-Kit, NCAM and Chromogranin A, in TNETs are detailed above.

WHO, World Health Organization; ***n(F:M)***, number(Female:Male);***TC***, Thymic Carcinoma; ***MNT***, Micronodular Thymoma; ***TNET***, Thymic Neuroendocrine Tumor; ***HSP 27***, Heat Shock Protein 27; ***HSP70***, Heat Shock Protein 70; ***Lu5***, Pancytokeratin; ***NCAM/CD56***, Neural Cell Adhesive Molecule; ***Chromo***, Chromogranin A; ***Pax8***, Paired

box protein 8; ***CK5/6***, Cytokeratin 5/6. **+/-/n.p.**, positive/negative/not performed. *one case was excluded because of tissue quality

Supplementary Table 2

Clinicopathologic characteristics		HSP expression in cells of the tumor microenvironment							
		cyto HSP27 expression (mean±SEM)							
		n	*ECs*	*n*	*Fibro*	*n*	*Adipo*	*n*	*Macro*
**WHO**	A	15	2.73±0.1	16	2.16±0.2	13	1.88±0.2	14	1.93±0.2
	AB	14	2.89±0.1	14	2.29±0.1	13	2.08±0.2	9	2.44±0.2
	B1	9	2.89±0.1	9	2.50±0.3	9	1.61±0.3	7	2.29±0.3
	B2	21	2.76±0.1	21	2.05±0.1	20	1.75±0.1	15	2.27±0.1
	B3	17	2.79±0.1	17	1.97±0.2	16	1.59±0.2	15	2.23±0.1
	TC	15	2.77±0.1	15	2.47±0.1	13	2.04±0.2	10	2.55±0.2
	MNT	4	2.75±0.3	4	2.0±0.4	3	2.17±0.2	0	-
	TNET	3	3.0±0.0	3	2.83±0.2	3	1.67±0.3	2	2.0±0.0
p-value			0.879[a]		0.137[a]		0.300[a]		0.321[a]
**Masaoka Koga Stage**	I	23	2.74±0.1	23	2.22±0.1	21	1.74±0.1	18	2.33±0.1
	II	46	2.85±0.0	46	2.16±0.1	43	1.88±0.1	31	2.27±0.1
	III	14	2.68±0.1	15	2.03±0.2	13	2.0±0.2	11	2.09±0.2
	IV	15	2.87±0.1	15	2.57±0.2	13	1.62±0.2	12	2.21±0.2
p-value			0.343[a]		0.119[a]		0.355[a]		0.784[a]
**MG**	Pos.	29	2.88±0.1	29	2.16±0.1	28	1.70±0.1	23	2.43±0.1
	Neg.	69	2.77±0.0	70	2.24±0.1	62	1.89±0.1	49	2.16±0.1
p-value			0.126[b]		0.583[b]		0.181[b]		0.090[b]
		cyto HSP70 expression (mean±SEM)							
		n	*ECs*	*n*	*Fibro*	*n*	*Adipo*	*n*	*Macro*
**WHO**	A	15	0.93±0.2	16	1.03±0.2	13	0.77±0.2	13	1.23±0.2
	AB	14	1.32±0.2	14	1.71±0.2	12	0.79±0.2	9	2.28±0.2
	B1	10	0.65±0.2	10	1.20±0.3	10	0.40±0.2	8	2.19±0.2
	B2	21	1.40±0.2	21	1.12±0.2	20	0.40±0.1	15	1.53±0.2
	B3	17	0.91±0.1	17	0.35±0.1	16	0.31±0.1	16	1.50±0.2
	TC	15	0.87±0.2	15	1.20±0.2	13	0.69±0.2	13	1.38±0.3
	MNT	4	0.63±0.1	4	1.0±0.5	3	0.67±0.4	0	-
	TNET	3	1.33±0.3	3	1.17±0.2	3	0.33±0.3	2	2.0±0.5
p-value			0.106[a]		0.001[a]		0.366[a]		0.007[a]
**Masaoka Koga Stage**	I	23	1.11±0.2	23	1.26±0.2	20	0.55±0.2	18	1.81±0.2
	II	47	1.03±0.1	47	0.98±0.1	44	0.45±0.1	32	1.66±0.1
	III	14	1.36±0.2	14	1.33±0.2	13	0.88±0.2	14	1.43±0.2
	IV	15	0.70±0.2	15	0.83±0.2	12	0.46±0.1	12	1.46±0.1
p-value			0.156[a]		0.188[a]		0.215[a]		0.476[a]
**MG**	Pos.	29	1.16±0.2	29	0.98±0.2	27	0.44±0.1	22	1.57±0.2
	Neg.	70	1.0±0.1	71	1.11±0.1	63	0.58±0.1	54	1.64±0.1
p-value			0.447[b]		0.522[b]		0.376[b]		0.718[b]

Suppl. Table 2. HSP expression in cells of the tumor microenvironment. HSP27 and 70 were expressed in ECs, fibroblasts, adipocytes and macrophages but were missing in lymphocytes. Strongest HSP27 staining was found in ECs. Neither Masaoka-

Koga tumor stage nor paraneoplastic MG had influence on staining intensity of cells in the tumor microenvironment. ***HSP27***, Heat Shock Protein 27; ***HSP70***, Heat Shock Protein 70; n, number; ***cyto*,** cytoplasmic expression; ***WHO***, World Health Organization; ***TC*,** Thymus Carcinoma; ***MNT***, Micronodular Thymoma**;** ***TNET*,** Thymic Neuroendocrine Tumor; ***MG***, Myasthenia Gravis**;** ***ECs*,** Endothelial Cells; ***Fibro***, Fibroblasts; ***Adipo*,** Adipocytes; ***Macro***, Macrophages; ***SEM***, standard error of the mean; [a] **one-way Anova**, [b] **independent-samples t-test.**

Supplementary Table 3

Characteristics						HSP expression in cells of non-malignant thymic specimens													
						HSP27							HSP70						
Case	***Group***	***Diagnosis***	***Sex***	***Age***	***MG***	*Lym*	*HC*	*mTEC*	*cTEC*	*GC*	*MZ*	*ECs*	*Lym*	*HC*	*mTEC*	*cTEC*	*GC*	*MZ*	*ECs*
**1**	Fetal	Reg. Thy.	F	20w	-	-	+	+	+	n.f.	n.f.	+	-	+	+/+	+/+	n.f.	n.f.	+
**2**	Fetal	Reg. Thy.	F	22w	-	-	+	+	+	n.f.	n.f.	+	-	+	+/+	+/+	n.f.	n.f.	+
**3**	Fetal	Reg. Thy.	M	22w	-	-	+	+	+	n.f.	n.f.	+	-	+	+/+	+/+	n.f.	n.f.	+
**4**	Infantil	Reg. Thy.	F	1a	-	-	+	+	+	n.f.	n.f.	+	-	+	+/+	+/+	n.f.	n.f.	+
**5**	Infantil	Reg. Thy.	F	1a	-	-	+	+	+	n.f.	n.f.	+	-	+	+/+	+/+	n.f.	n.f.	+
**6**	Infantil	Reg. Thy.	F	14a	-	-	+	+	+	n.f.	n.f.	+	-	+	+/+	+/+	n.f.	n.f.	+
**7**	Adult	TTH	F	40a	+	-	+	+	+	n.f.	n.f.	+	-	+	+/+	+/+	n.f.	n.f.	+
**8**	Adult	TTH	M	40a	-	-	+	+	+	n.f.	n.f.	+	-	+	+/+	+/+	n.f.	n.f.	+
**9**	Adult	Reg. Thy.	F	35a	+	-	+	+	+	n.f.	n.f.	+	-	+	+/+	+/+	n.f.	n.f.	+
**10**	Adult	TTH	F	27a	+	-	+	+	+	n.f.	n.f.	+	-	+	+/+	+/+	n.f.	n.f.	+
**11**	Adult	Reg. Thy.	F	34a	+	-	+	+	+	n.f.	n.f.	+	-	+	+/+	+/+	n.f.	n.f.	+
**12**	Adult	TTH	M	30a	-	-	+	+	+	n.f.	n.f.	+	-	+	+/+	+/+	n.f.	n.f.	+
**13**	Adult	TTH	M	48a	-	-	+	+	+	n.f.	n.f.	+	-	+	+/+	+/+	n.f.	n.f.	+
**14**	Adult	TTH	F	26a	-	-	+	+	+	n.f.	n.f.	+	-	+	+/+	+/+	n.f.	n.f.	+
**15**	Adult	TTH	F	21a	-	-	+	+	+	n.f.	n.f.	+	-	+	+/+	+/+	n.f.	n.f.	+
**16**	Adult	TTH	M	57a	-	-	+	+	+	n.f.	n.f.	+	-	+	+/+	+/+	n.f.	n.f.	+
**17**	Adult	TTH	M	55a	-	-	+	+	+	n.f.	n.f.	+	-	+	+/+	+/+	n.f.	n.f.	+
**18**	Adult	TTH	M	72a	-	-	+	+	+	n.f.	n.f.	n.f.	-	+	+/+	+/+	n.f.	n.f.	n.f.
**19**	Adult	FTH	F	22a	+	-	+	+	+	+	+	+	-	+	+/+	+/+	+/+	+/+	+
**20**	Adult	FTH	F	29a	+	-	+	+	+	+	+	+	-	+	+/+	+/+	+/+	+/+	+
**21**	Adult	FTH	M	29a	+	-	+	+	+	+	+	+	-	+	+/+	+/+	+/+	+/+	+
**22**	Adult	FTH	M	15a	+	-	+	+	+	+	+	+	-	+	+/+	+/+	+/+	+/+	+
**23**	Adult	FTH	F	17a	+	-	+	+	+	+	+	+	-	+	+/+	+/+	+/+	+/+	+
**24**	Adult	FTH	M	19a	+	-	+	+	+	+	+	+	-	+	+/+	+/+	+/+	+/+	+

Suppl. Table 3. HSP27 and HSP70 expression in thymic physiology. HSPs were expressed in all medullary and cortical TECs, HCs and ECs. In myasthenic patients with FTH HSPs were expressed in DCs of GCs and in TECs with marginal zone like distribution. ***HSP27***, Heat Shock Protein 27; ***HSP70***, Heat Shock Protein 70; ***F***, Female; ***M***, Male,***w***, age in weeks of pregnancy; ***a*,** age in years; ***Reg. Thy.,***Regular Thymus for age; ***TTH,*** True Thymic Hyperplasia; ***FTH***; Follicular Thymic Hyperplasia; ***MG***, Myasthenia Gravis; ***Lym***, Lymphocytes; ***HC***, Hassall's Corpuscles; ***mTEC***, medullary Thymic Epithelial Cells; ***cTEC***, cortical Thymic Epithelial Cells; ***GC***, Germinal Center;

MZ, TECs with marginal zone like distribution; ***EC***, Endothelial Cells. **+**, positive staining; **-**, negative staining; **n.f.,** not found; **+/+**, positive nuclear and cytoplasmic staining.

Supplementary Table 4

Masaoka-Koga Stage	n	HSP serum concentrations			
		HSP27 [pg/ml]		HSP70 [ng/ml]	
		mean(median)±SD(SEM)	*p-value*	*mean(median)±SD(SEM)*	*p-value*
I-II	22	358.9(267.4)±367.8(78.4)		1.6(1.0)±1.4(0.3)	
III-IV	24	647.8(429.4)±491.7(100.4)	0.028[b]	2.4(1.9)±1.9(0.4)	0.111[b]
Invasive (II-IV)	39	521.0(340.8)±449.2(71.9)		2.1(1.7)±1.8(0.3)	
Non-Invasive (I)	7	446.2(285.0)±525.8(198.7)	0.789[b]	1.4(1.0)±1.0(0.4)	0.203[b]
Local (I-III)	28	426.9(311.3)±407.1(76.9)		1.7(1.0)±1.6(0.3)	
Metastasis (IV)	18	638.3(412.0)±508.5(119.9)	0.148[b]	2.4(1.9)±1.9(0.4)	0.203[b]

Suppl. Table 4. Further stratification of HSP27 and 70 serum concentrations in patients with TETs according to Masaoka-Koga Stage. ***n***, number; ***HSP27***, Heat Shock Protein 27; ***HSP70***, Heat Shock Protein 70; ***TETs***, Thymic Epithelial Tumors;

[b] independent-samples t-test.

Supplementary Figure S1

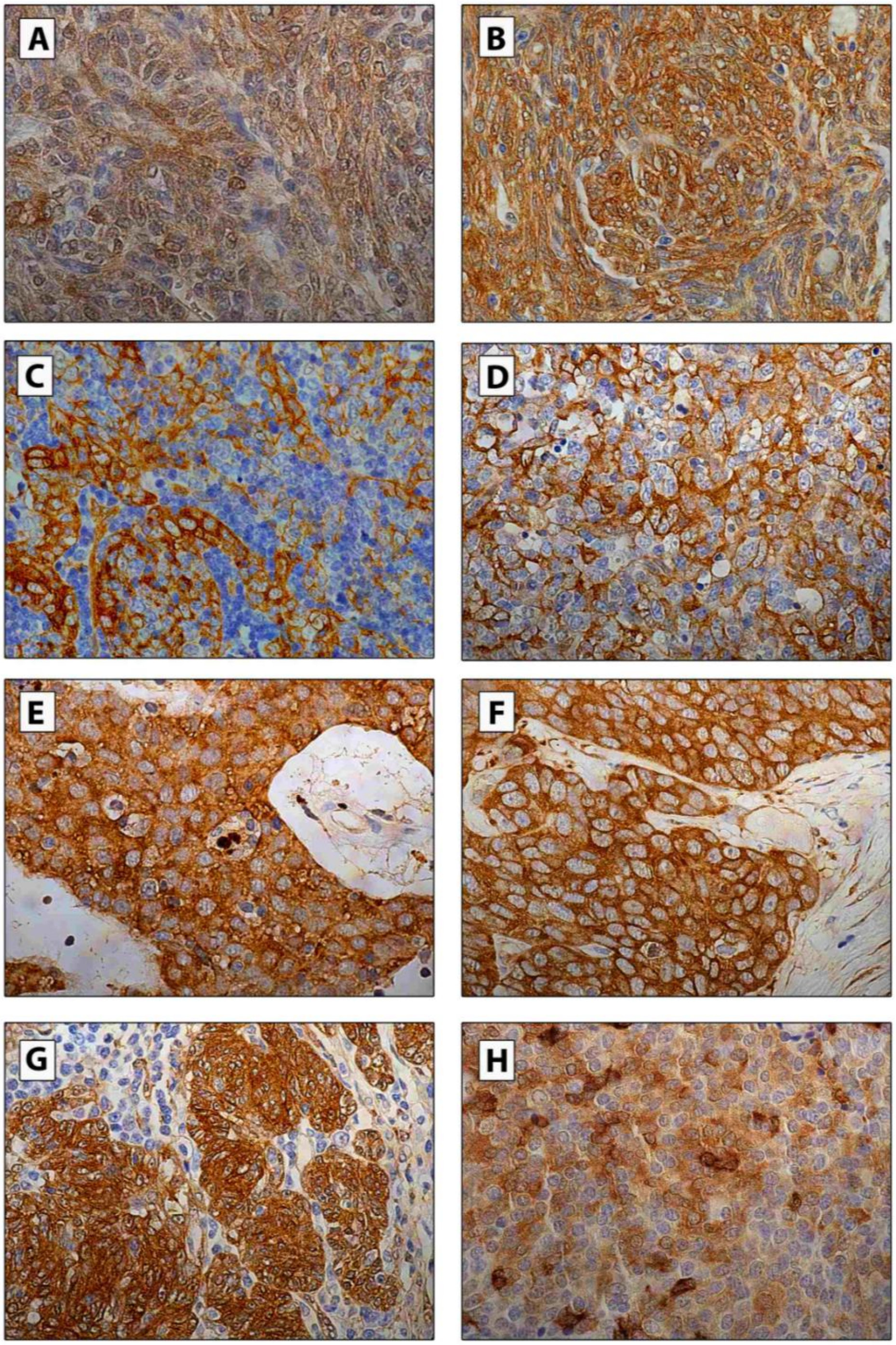

Supplementary Fig.1. HSP27 expression in TETs. Immunohistochemical staining of HSP27 in different histologic tumor subtypes was performed. Expression of HSP27 in thymoma WHO type A (**A**), A-component type AB (**B**), B1admixed with B2 (**C**), B2 (**D**),

B3 (**E**), TC - SCC (**F**), MNT (**G**) and TNET (**H**) is shown *(630x magnification).*Hematoxylin was used for counterstaining***. HSP27,*** Heat Shock Protein 27; ***TETs,*** Thymic Epithelial Tumors;***WHO,*** World Health Organization; ***TC***, Thymic Carcinoma; ***SCC,*** Squamous Cell Carcinoma; ***MNT***, Micronodular Thymoma; ***TNET***, Thymic Neuroendocrine Tumor.

Supplementary Figure S2

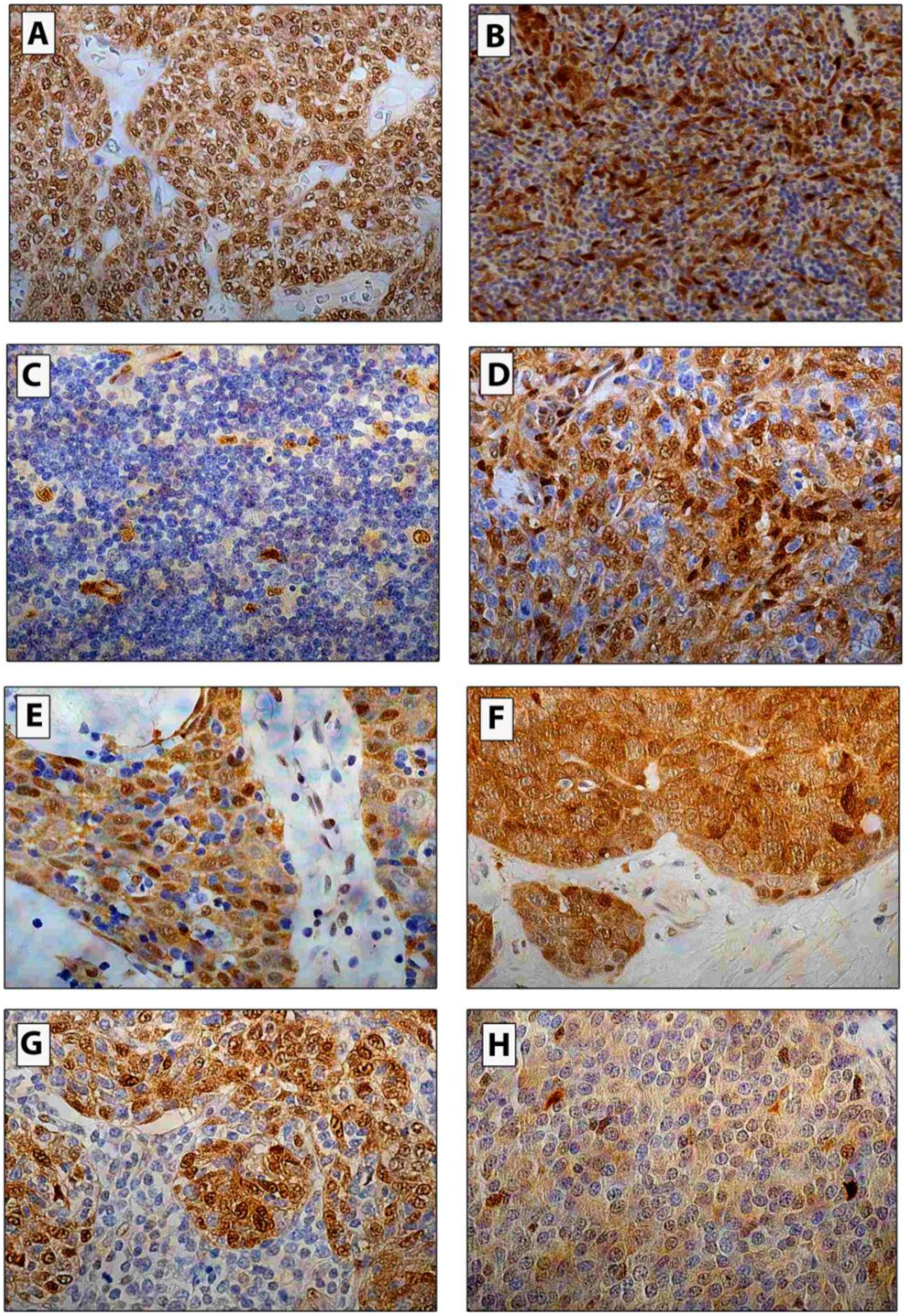

Supplementary Fig.2. HSP70 expression in TETs. Immunohistochemical staining of HSP70 in different histologic tumor subtypes was performed. Expression of HSP70 in thymoma WHO type A (**A**), A-component type AB(**B**), B1 (**C**), B2 (**D**), B3 (**E**), TC - SCC

(**F**), MNT (**G**) and TNET (**H**) is shown *(630x magnification).*Hematoxylin was used for counterstaining***. HSP70****,* Heat Shock Protein 70; ***TETs****,* Thymic Epithelial Tumors;***WHO****,* World Health Organization; ***TC***, Thymic Carcinoma; ***SCC****,* Squamous Cell Carcinoma; ***MNT***, Micronodular Thymoma; ***TNET***, Thymic Neuroendocrine Tumor.

Supplementary Figure S3

Epithelial cells that undergo malignant transformation retain their physiologic keratin profiles (1). Cytokeratins are helpful to distinguish TETs from other common anterior mediastinal tumors, such as lymphomas or germ cell tumors (1,2).

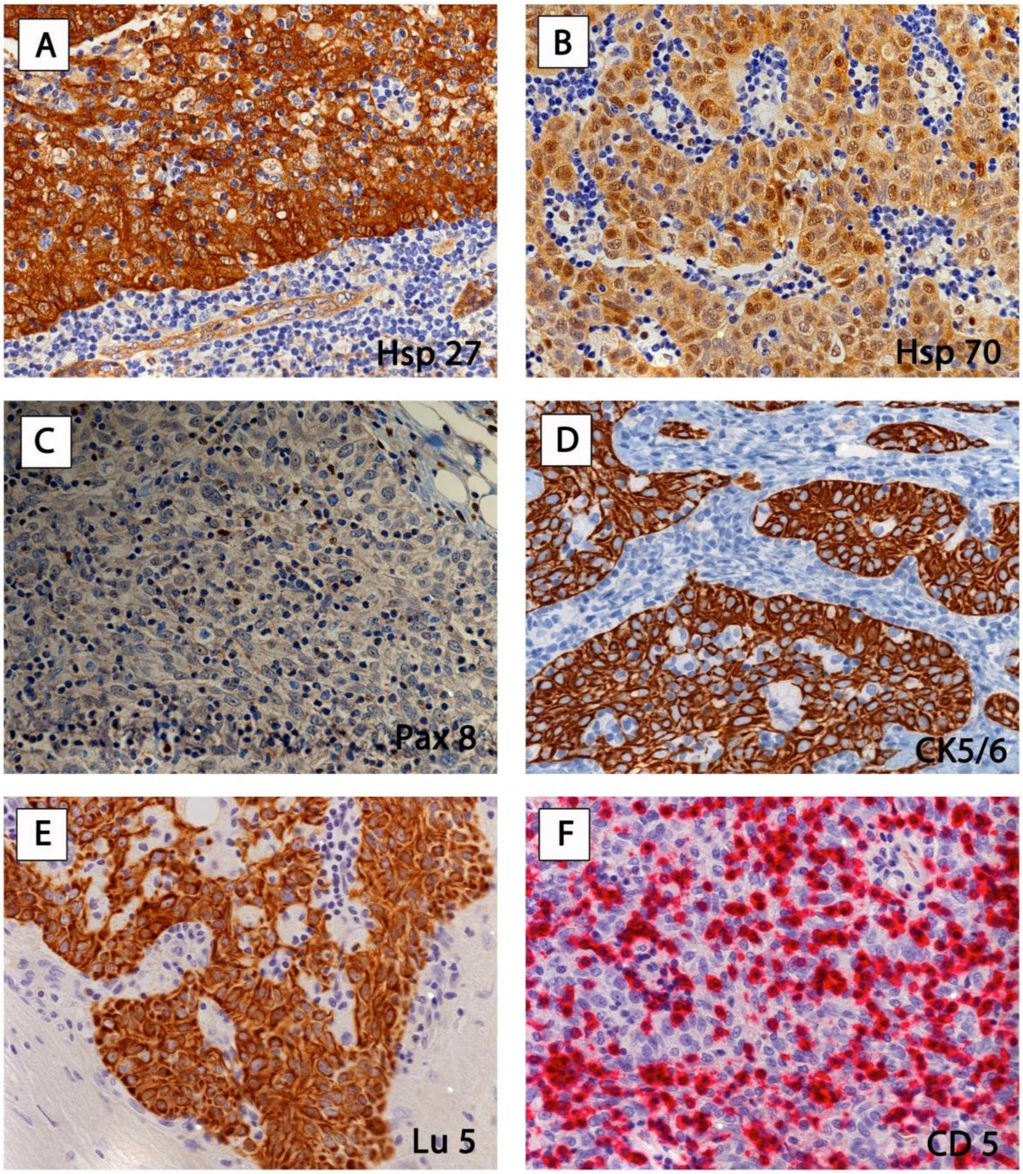

Supplementary Fig.3. Selected immunohistochemical markers used for routine diagnosis of TETs. Cytokeratins 5/6 are generally expressed in TECs and indeed 60-100% of TETs express CK5/6. Lu5/pancytokeratin mouse monoclonal antibody detects both types (acidic and basic) of cytokeratin subfamilies In our study CK5/6 and Lu5/pancytokeratin were expressed in 87.5% and 100% of TETs, respectively. In comparison HSP27 and 70 were expressed in 100% of TETs but either absent or weakly expressed in TNETs. Expression of HSP27 (**A**), HSP70 (**B**), Pax 8 (**C**), CK5/6 (**D**), Lu 5 (**E**) and CD5 (**F**) in WHO type B2 thymoma is shown (400x magnification). ***HSP27,*** Heat Shock Protein 27; ***HSP70,*** Heat Shock Protein 70; ***Pax 8,*** Paired box protein 8; ***CK5/6,*** Cytokeratin 5/6; ***Lu 5,*** pancytokeratin.

The following antibodies were used: monoclonal mouse anti-human Lu-5 (pancytokeratin) IgG1 (Biocare Medical, Concord, CA, USA)monoclonal mouse anti-human CD5 IgG1κ(Clone 4C7, LEICA (NovoCastra), Nussloch, Germany), monoclonal mouse anti-human CD20 IgG2α (Clone L26, DAKO, Glostrup, Denmark), monoclonal mouse anti-human CD56/NCAM IgG1 (Clone 1B6, LEICA (NovoCastra), Nussloch, Germany), polyclonal rabbit anti-human CD117/c-Kit (DAKO, Glostrup, Denmark), monoclonal mouse anti-human CK5/6 (Clone D5/16B4, EUBIO, Wien, Austria), monoclonal mouse anti-human Chromogranin A (Clone 5H7, LEICA(NovoCastra), Nussloch, Germany) and polyclonal rabbit anti-human PAX8 (EUBIO, Wien, Austria).

Supplementary Information about antibody specificity to HSP27 and 70

A set of rules was used to select antibodies to HSP27 and 70 (4). The antibodies were raised against published antigens and tested on whole cell lysates. The monoclonal HSP70/HSPA1A Antibody (W27) was tested by western blot on transfected A549 cells (5). The monoclonal HSP 27 (F-4) antibody was tested by western blot analysis of HSP 27 expression in mouse HSP 27 transfected 293T and ECV304 whole cell lysates (6).

Supplementary References

(1) Chu, P. G. &Weiss,L. M. Keratin expression in human tissues and neoplasms. *Histopathology***40,** 403-439 (2002).

(2) Chu, P. G. &Weiss, L. M. Expression of cytokeratin 5/6 in epithelial neoplasms. *Mod Pathol***15,** 6-10 (2002).

(3) Schröder, S., Wodzynski, A. &Padberg, B. Cytokeratin expression of benign and malignant epithelial thyroid gland tumors. An immunohistologic study of 154 neoplasms using 8 different monoclonal cytokeratin antibodies. *Pathologe*. **17**(6)**,** 425-32 (1996).

(4) Saper, C. P. A guide to the perplexed on the specificity of antibodies. *J Histochem Cytochem*. **57**(1), 1-5 (2009).

(5) http://www.novusbio.com/PDFs/NB600-571.pdf (11.02.2016)

(6) http://datasheets.scbt.com/sc-59562.pdf (11.02.2016)
